# Mechanistic insights into the interaction between the host gut microbiome and malaria

**DOI:** 10.1371/journal.ppat.1011665

**Published:** 2023-10-12

**Authors:** Rabindra K. Mandal, Nathan W. Schmidt

**Affiliations:** Ryan White Center for Pediatric Infectious Diseases and Global Health, Herman B Wells Center for Pediatric Research, Department of Pediatrics, Indiana University School of Medicine, Indiana, United States of America; Joan and Sanford I Weill Medical College of Cornell University, UNITED STATES

## Abstract

Malaria is a devastating infectious disease and significant global health burden caused by the bite of a *Plasmodium*-infected female *Anopheles* mosquito. Gut microbiota was recently discovered as a risk factor of severe malaria. This review entails the recent advances on the impact of gut microbiota composition on malaria severity and consequence of malaria infection on gut microbiota in mammalian hosts. Additionally, this review provides mechanistic insight into interactions that might occur between gut microbiota and host immunity which in turn can modulate malaria severity. Finally, approaches to modulate gut microbiota composition are discussed. We anticipate this review will facilitate novel hypotheses to move the malaria-gut microbiome field forward.

## 1. Introduction

Malaria is an infectious disease caused by the bite of a female *Anopheles* mosquito infected with the parasite *Plasmodium*. Malaria remains a significant burden on the global healthcare system, causing more than 627,000 deaths and 241 million cases in 2020 [1,2]. Greater than 90% of infections and severe malaria in humans is caused by *P*. *falciparum*, with additional infections caused by other *Plasmodium* species including *P*. *vivax*, *P*. *malaria*, *P*. *ovale*, and *P*. *knowlesi* [3]. Sequestration of infected RBCs in internal organs lead to widespread organ damage and is a major cause of death in patients with severe *P*. *falciparum* malaria [4,5]. However, additional factors that determine the severity of malaria in humans are still evolving. Recently, it was shown that the gut microbiome (i.e., microorganism including their genetic content, microbial products, and environment within the gut) is a risk factor of severe malaria [6,7].

The phrase “you are what you eat” which can be extended to “what you eat is your gut microbiome” is very true in the context of gut microbiome that play a critical role in health and disease of individuals [8–10]. Bacteria make up the major organic fraction of feces (approximately 25% to 54% of dry solids) in humans [11]. Approximately 70% to 80% of immune cells are located in the gut that are trained by the intestinal microbiome [12]. Innate and adaptive immunity are required to control *Plasmodium* infection. Thus, gut microbiota that influence local and systemic immune system have the potential to significantly impact antimalarial immunity [13–17].

Here, we have provided mechanistic insight into the role of gut microbiome in shaping malaria severity, including severe malaria anemia (SMA) and cerebral malaria. The presence of specific gut bacteria like *Ruminococcaceae*, *Lachnospiraceae*, *Bacteroides*, and *Blautia* were associated with severe malaria while *Bifidobacterium* and *Escherichia* are correlated with better outcome following *Plasmodium* infection. In addition to preinfection bacteria composition correlating with malaria outcomes, bacteria populations have also been shown to change in abundance following infection. Bacterial genera including *Bacteroides*, *Alistipes*, and *Clostridia* are relatively increased while *Ruminococcaceae*, *Prevotellaceae*, and *Ruminococcus* are decreased in abundance during *Plasmodium* infection. Importantly, gut bacteria represent a druggable target to boost antimalarial immunity and decrease malaria severity.

## 2. Associations between human gut microbiome and malaria

**Fig 1:**
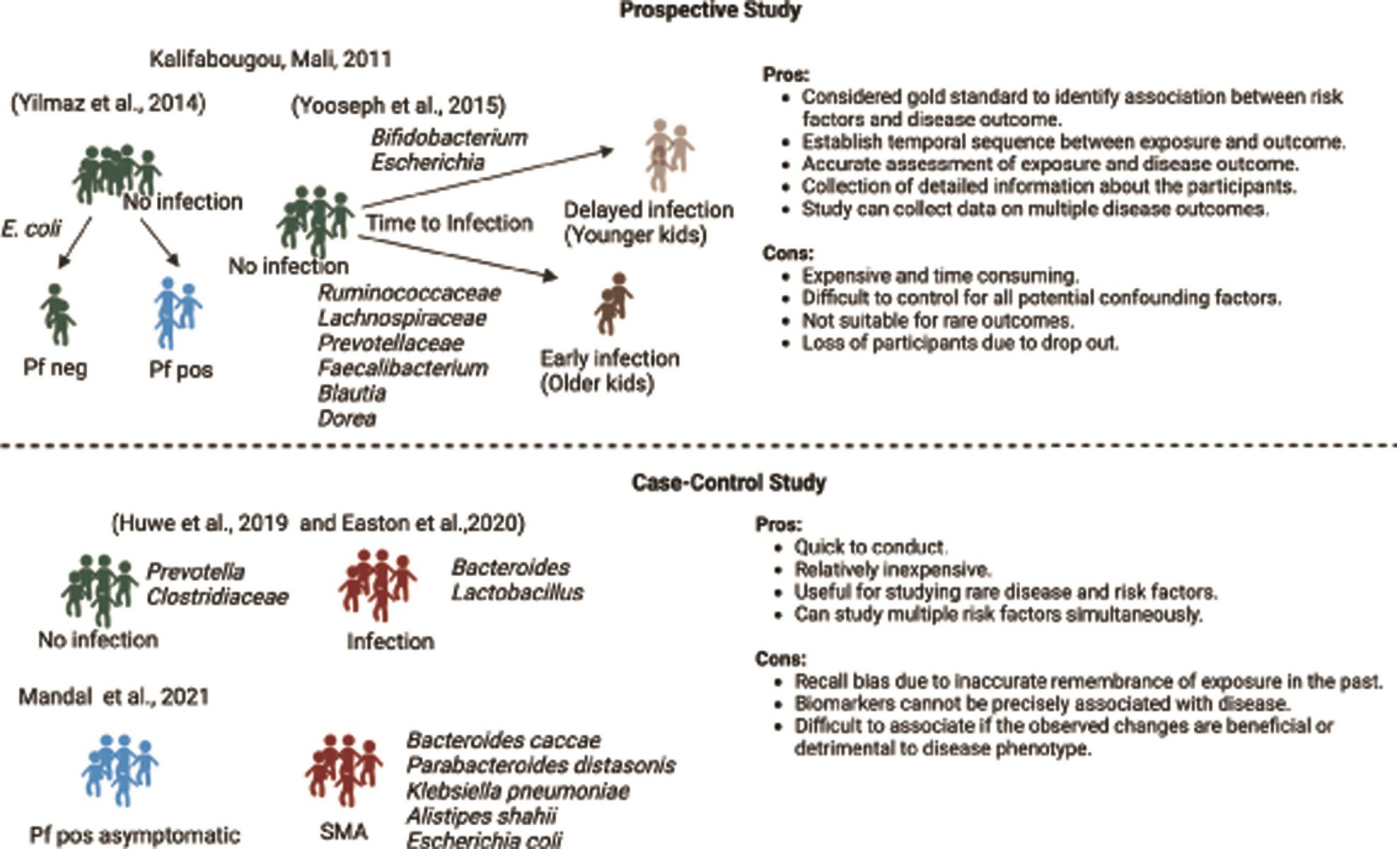
Gut bacteria associated with human malaria. To date 5 peer-reviewed studies have been published on the impact of the gut microbiota in *Plasmodium* infection in humans. A cohort of children in Kalifabougou, Mali was used for 2 prospective studies (Yilmaz and colleagues (2014) [18] and Yooseph and colleagues (2015) [28]). Additionally, 3 case-control studies investigated association of gut microbiota at the time of *Plasmodium* infection (Huwe and colleagues (2019) [32] and Easton and colleagues (2020) [33]) and SMA (Mandal and colleagues (2021) [6]). Prospective and case-control studies have their own advantages and disadvantages. Bacteria associated with the clinical outcome is shown. Pf: *P. falciparum*, neg: negative, pos: positive. Figure was created with BioRender.com.

In 2014, Yilmaz and colleagues showed that older individuals (4 to 25 years old) with higher levels of Galα1-3Galβ1-4GlcNAc-R (α-gal) glycan-specific IgM antibodies in plasma were significantly protected against *P*. *falciparum* infection [18]. However, younger children (3 months to 4 years old) had no association between protection from *P*. *falciparum* infection and α-gal IgM antibodies in the plasma. Evolutionarily, humans do not express α-gal, allowing them to generate anti-α-gal antibodies when colonized by α-gal expressing bacteria [19]. Other microorganisms, including *Plasmodium*, express α-gal, providing an opportunity for cross-reaction between bacterial-induced anti-α-gal antibodies and other microbes. The authors further confirmed the role of α-gal produced by *Escherichia coli* O86:B7, a member of gut microbiota, in the mouse model of malaria (Fig 1). Anti-α-gal IgM antibodies confer protection against *Plasmodium* sporozoite infection independent of complement induced polymorphonuclear leukocyte recruitment, but rather by complement-induced cytotoxicity. Anti-α-gal IgM antibodies also conferred protection, in part, via blocking sporozoite migration and infection of hepatocytes, thereby blocking development of exoerythrocytic forms within hepatocytes. In contrast, anti-α-gal antibodies had no effect on the erythrocytic stage of infection in mice [18]. Additionally, in an RTS,S/AS0 vaccination trial conducted in Mozambique and Ghana, vaccinated infants (1.5 to 3 months) who had higher level of anti-α-gal IgM plasma antibodies were protected against clinical malaria over a one-year follow up compared to infants with low levels of IgM plasma antibody [20]. Nonetheless, anti α-gal IgM levels were not associated with protection against malaria in vaccinated older children (5 to 17 months) [20].

Multiple gut bacteria can produce α-gal including specific members of *Klebsiella* spps., *Serratia* spp., and *Escherichia coli* spps. Likewise, lactic acid bacteria (LAB) like *Limosilactobacillus fermentum*, *Levilactobacillus brevis*, *Agrilactobacillus composti*, *Lacticaseibacillus paracasei, Leuconostoc mesenteroides*, and *Weissella confuse* can express α-gal [13]. Probiotic bacteria *Aeromonas veronii* and *Pseudomonas entomophila* have high α-gal content [21]. Additionally, pathogenic bacteria including *Salmonella* spp. Beyond bacteria, *Trypanosoma* spp., *Aspergillus fumigatus*, and *Leishmania* spps. can produce α-gal [22–25]. Of note, *Klebsiella* spps. and *Escherichia* are reported to be associated with malaria severity [6]. Although anti-α-gal antibodies were associated with protection from pre-erythrocytic stages of infection, they provided no benefit against blood-stage infection [18]. Therefore, it is possible that while bacteria-induced anti-α-gal antibodies confer protection against pre-erythrocytic stages, these same bacteria (e.g., *Klebsiella* spps. and *Escherichia*) acting through different mechanisms could also contribute to severe blood-stage infections. On the other hand, *Trypanosoma brucei* infection protects mice against experimental cerebral malaria in a coinfection model [26]. However, it’s not clear if trypanosomiasis protects against malaria in human [27].

A study by Yooseph and colleagues in 2015 [28] (using the same prospective cohort of Malian children as used by Yilmaz and colleagues (2014) [18]) reported that younger children (average age of 1.4 years) had a delayed time to first *P*. *falciparum* infection with median 121 days (95% CI 101 to 150) compared to older children (average age of 9.1 years) with median 85 days (95% CI 73 to 99). By accounting for age, gender, anemia, HbAS, *S*. *hematobium* infection, splenomegaly, and distance to river, the authors concluded that younger children stool microbiota significantly protected against prospective risk of *P*. *falciparum* infection compared to stool microbiome in older children. However, stool microbiota did not correlate with protection against febrile malaria episodes between the young and old children. Older children with increased prospective risk and shorter time to *P*. *falciparum* infection had significantly higher abundance of *Ruminococcaceae* unclassified, Lachnospiraceae unclassified, *Prevotellaceae* unclassified at family level; and *Faecalibacterium*, *Blautia*, and *Dorea* at genus level compared to younger children. Young children with delayed *P*. *falciparum* infection had significantly higher relative abundance of *Bifidobacterium*, *Streptococcus*, *Escherichia/Shigella* compared to older children among others (Fig 1). It was also reported that the stool microbiota composition was different between children (average age 3.2 years) who had a persistent asymptomatic *P*. *falciparum* infection carried over from the previous malaria transmission season than children (average age 1.3 years) who had no *P*. *falciparum* infection at the end of six-month dry season before start of subsequent six-month malaria season in Mali. This result may imply that gut microbiota composition in dry season might be involved in persistence or asymptomatic *P*. *falciparum* infection.

A limitation of Yilmaz and colleagues (2014) [18] towards our understanding into how gut microbiota impact *Plasmodium* infections and severity of malaria is that the authors did not identify the source of anti-α-gal IgM antibodies in the participants or if the abundance of *E*. *coli* O86:B7 in stool is associated with protection against *P*. *falciparum* infection. Likewise, Yooseph and colleagues (2015) [28] did not account for baseline anti-α-gal IgM antibody levels or if they are associated with time to delayed *P*. *falciparum* infection in younger kids during gut microbiota analysis. Thus, it’s not clear if the delayed *P*. *falciparum* infection in younger kids (1.4 years) compared to older kids is due to cross-reactivity between anti-α-gal IgM antibodies and *P*. *falciparum* or with differential gut microbiota composition. Noteworthy, Yooseph and colleagues (2015) [28] reported higher abundance of *Escherichia/Shigella* in the stool of younger kids with delayed protection from *P*. *falciparum* infection. Additionally, in the same Malian cohort *P*. *falciparum* reticulocyte-binding protein homologue 5 (PfRH5) specific IgG antibody was associated with a longer time to blood-stage infection and first febrile malaria and enhanced p53 expression in monocytes was predicted to be protective against febrile malaria [29,30]. Finally, *P*. *falciparum* Schizont Egress Antigen-1 anti-(PfSEA-1) in plasma is associated with a decreased risk of severe malaria in 1.5 to 4 years old kids in a holoendemic area of Tanzania [31]. These observations highlight the complexity of human malaria outcomes and the challenge to account for these while assessing the contribution of gut microbiota as a risk factor.

A study by Huwe and colleagues (2019) [32] in India where soil-transmitted helminths and malaria are endemic, reported a significantly higher abundance of *Lactobacillus* genus in the stool of individuals aged 0 to 68 years old infected with *P*. *falciparum* and *P*. *vivax* compared to noninfected individuals (Fig 1). Within this cohort (*n* = 68), 46% were infected with *Plasmodium* [32]. In another study, Easton and colleagues (2020) [33] found that stool bacterial taxa of Colombian children aged 4 to 16 years were stronger predictors of *P*. *vivax* parasitemia levels compared to the host peripheral blood transcriptome response or complete blood count. Although principal component analysis showed that overall gut microbiota composition was not significantly different between *P*. *vivax*-infected and uninfected children, differential abundance analysis identified higher prevalence of stool *Bacteroides* and lower abundance of *Prevotella* and *Clostridiaceae* were associated with *P*. *vivax* infection compared to uninfected individuals (Fig 1). A critical limitation of these studies is the comparison of gut bacteria between *Plasmodium*-infected and uninfected children because it is not possible to know what the malaria outcomes would be in the uninfected children if they, too, were infected with *Plasmodium*. This limitation is addressed in a study that compared gut bacteria between Ugandan children (0.5 to 4 years old) with an asymptomatic *P*. *falciparum* infection to children with severe malarial anemia (SMA) [6]. Children with SMA had significantly different gut bacteria composition compared to children that had asymptomatic *P*. *falciparum* infection. Higher abundance of stool bacteria including *Escherichia coli*, *Parabacteroides distasonis*, *Bacteroides caccae*, and *Klebsiella pneumoniae* among others were predictive of SMA (Fig 1). The possible mechanism by which gut bacteria may mediate immunity to malaria is described in later section. Limitations of this study include the small sample size (*n* = 7) of the Ugandan children with asymptomatic *P*. *falciparum* infection and the use of case-controls as opposed to a longitudinal prospective study. The latter limitation is potentially important as *P*. *falciparum* may cause changes in gut bacteria (discussed below).

## 3. Host gut microbiome impacts pathogenesis of malaria—Lessons from nonhuman vertebrate models

Several publications have reported the association between gut microbiota composition and malaria outcomes in mouse malaria models. Since 2016, it has been shown in several studies that baseline gut bacteria structure, function, and composition dictate susceptibility to *P*. *yoelii* 17XNL hyperparasitemia in specific-pathogen-free (SPF) C57BL/6 and BALB/c mice from different vendors and isolated barrier units (IBUs) [6,34–39]. These outcomes are not restricted to mice from different vendors and IBUs, as antibiotic-induced changes in gut microbiota, both before *P*. *yoelii* 17XNL and up to 7 days after *P*. *yoelii* 17XNL infection can impact severity of malaria [6]. Additionally, in outbred Swiss Webster mice, gut microbiota composition determined *P*. *chabudi chabudi* AS infection pregnancy malaria outcomes [40]. Gut microbiota is a consortium of bacteria, fungi, archaea, viruses, and helminths. Among these microbes, it was shown that gut bacteria modulate the severity of *P*. *yoelii* 17XNL hyperparasitemia [6]. However, the potential contribution of other members of gut microbiota (e.g., fungi, archaea, and helminth) cannot be overlooked. Indeed, intestinal helminths are known to modulate malaria outcomes mostly exacerbating *P*. *falciparum* and *P*. *vivax* infection, yet the effects of intestinal helminths on malaria outcomes remains controversial [33,41–44].

**Fig 2:**
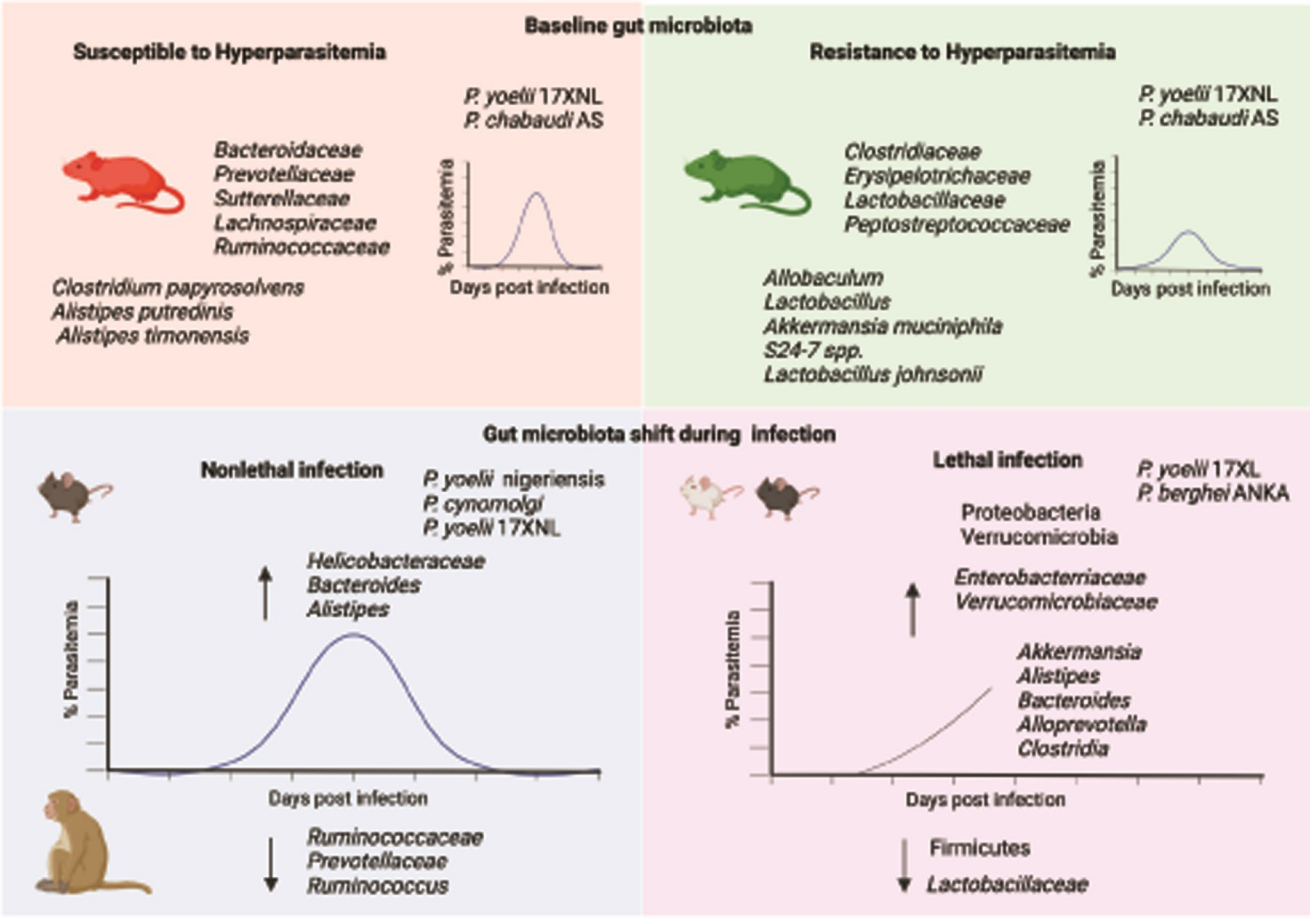
Gut bacteria associated with nonhuman model of malaria. Top 2 panels show baseline gut bacteria differentially abundant in mice either susceptible or resistant to *P*. *yoelii* 17XNL hyperparasitemia and *P*. *chabaudi* AS pregnancy outcomes. Fecal pellets are collected at baseline prior to *Plasmodium* infection to determine the gut microbiota composition. Bottom 2 panels show the shift in gut microbiota composition during *Plasmodium* infection in mice and monkeys. In nonlethal infection models, fecal pellet microbiota composition at peak parasitemia is compared to before *Plasmodium* infection. Bacteria that are significantly increased (up arrow) or decreased (down arrow) are shown. *P*. *yoelii* 17XL causes lethal infection due to hyperparasitemia while *P*. *berghei* ANKA causes mortality due to experimental cerebral malaria. Gut fecal samples are collected before mortality and compared to baseline gut microbiota. Changes in bacteria population at phylum, family, and genus level are shown. Figure was created with BioRender.com.

Gut bacteria could exert an effect on malaria outcomes by providing protection from severe malaria or by causing susceptibility to severe malaria. Presently, results from the *P*. *yoelii* 17XNL hyperparasitemia model suggest bacteria cause susceptibility, rather than resistance, to *P*. *yoelii* 17XNL hyperparasitemia. First, *P*. *yoelii* 17XNL hyperparasitemia-susceptible C57BL/6N mice treated with antibiotics have low parasitemia, while antibiotic-treated hyperparasitemia-resistant C57BL/6N mice were largely unaffected [6]. Second, fecal microbiota transplant (FMT) from hyperparasitemia-susceptible C57BL/6N mice➔hyperparasitemia-resistant C57BL/6N mice confers susceptibility while hyperparasitemia-resistant FMT➔hyperparasitemia-susceptible mice did not confer resistance [6]. Although these results favor gut bacteria can cause susceptibility to *P*. *yoelii* 17XNL hyperparasitemia, additional research in this model, and others, may identify protective roles of bacteria against severe malaria. C57BL/6N mice that are susceptible to severe hyperparasitemia have decreased numbers of germinal center (GC) B cells and follicular helper T (Tfh) cells and decreased titers of *P*. *yoelii* 17XNL-specific antibodies that recognize a smaller repertoire of *P*. *yoelii* 17XNL antigens [6,34]. C57BL/6N mice that develop *P*. *yoelii* 17XNL hyperparasitemia have significantly higher abundance of gut bacteria including *Bacteroidaceae*, *Prevotellaceae*, and *Sutterellaceae* at family level and *Clostridium papyrosolvens*, *Alistipes putredinis*, and *Alistipes timonensis* species compared to C57BL/6N mice resistant to *P*. *yoelii* 17XNL hyperparasitemia (Fig 2) [6,34].

Functionally, as revealed by ceca metatranscriptomics, mice susceptible to *P*. *yoelii* 17XNL hyperparasitemia had overexpression of *filC*, *ureABC*, and 6 members of *nuo* gene family related to gut microbes [36]. Overexpression of *filC*, which encodes bacterial flagellin, is associated with mucosal barrier breakdown and inflammation [45,46]. Short-chain fatty acids (SCFAs) are bacterial metabolites that have pleotropic effects on host health. SCFAs play a vital role in energy metabolism, immunologic homeostasis, and gut barrier integrity [47]. Only the level of propionic acid (PA) among 7 other SCFAs was significantly higher in mice susceptible to *P*. *yoelii* 17XNL hyperparasitemia [39]. Bacteroidetes, which are abundant in *P*. *yoelii* 17XNL hyperparasitemia susceptible mice [6], are able to ferment polysaccharides to PA [48]. *Bacteroides acidifaciens* in the mouse gut are able to significantly increase the level of SCFAs especially PA [49]. Higher level of PA in gut suppresses inflammation and ameliorates liver ischemia and reperfusion injury in mice. Increased levels of circulating PA is linked to higher cognitive decline in older persons [50] and associated with innate neuroinflammation, increased oxidative stress, glutathione depletion, and altered phospholipid/acylcarnitine profiles linked to autism spectrum disorder [48,51]. The degree to which SCFAs contribute to malaria outcomes, including cerebral malaria, are unknown.

*Plasmodium* species infect red blood cells (RBCs) and undergo asexual replication to produce merozoites or enter sexual differentiation to produce gametocytes. It is not known if gut microbiota composition impact RBC physiology. Intriguingly, functional profiling of whole ceca (ceca content + ceca tissue) metatranscriptomics of *P*. *yoelii* 17XNL hyperparasitemia-susceptible mice revealed increased expression of basigin, a cell surface receptor required for *P*. *falciparum* invasion of RBCs [36]. Recently, the gut bacteria *Flavonifractor plautii* was found to be involved in the conversion blood type A to universal O type blood [52]. Higher abundance of *Flavonifractor platutii* was seen in the Ugandan kids with severe malaria [6]. How gut bacteria impact the function of RBCs and *Plasmodium* biology within RBCs is an unexplored area.

## 4. Impact of *Plasmodium* infection on gut microbiota composition

### 4.1. Non-cerebral malaria models

While gut microbiota has been shown to impact malaria outcomes, research has also shown that *Plasmodium* infections can alter gut bacteria composition dependent on the murine model, parasite strain, malaria severity, and baseline gut microbiota. In C57BL/6 mice, *P*. *yoelii* nigeriensis infection increased gut *Bacteroides* and decreased *Ruminococcus* on day 10 post infection (Fig 2) [53]. Bacterial diversities were lowest (alpha index) on day 10 p.i. and recovered to baseline level by day 30 post infection [53]. A similar decrease in alpha diversity and increase in abundance of bacteria belonging to Bacteroidota (such as *Alistipes* and *Bacteroides*) were reported in BALB/c mice following lethal *P*. *yoelii* 17XL infection at day 5 post infection compared to baseline level (Fig 2) [54]. In a nonhuman primate model, rhesus macaques infected by *P*. *cynomolgi* have decreased gut microbial alpha diversity at the peak of infection with a dramatic increase in relative abundance of Proteobacteria (family *Helicobacteraceae*) while decrease in Firmicutes (family *Lactobacillaceae* and *Ruminococcaceae*), Bacteroidetes (family *Prevotellaceae*) (Fig 2) [55]. However, Denny and colleagues and Yawen and colleagues showed increase in alpha diversity in mice following *P*. *yoelii* 17XNL infection [38,56]. Mice that are resistant to *P*. *yoelii* 17XNL hyperparasitemia (peak parasitemia is below 20%) have increase in alpha diversity (observed OTUs). In contrast, alpha diversity was stable in *P*. *yoelii* 17XNL hyperparasitemia-susceptible mice (peak parasitemia reaches up to 60%) following infection. Beta diversity (Bray–Curtis distance) was impacted by *P*. *yoelii* 17XNL infection in both hyperparasitemia-susceptible and -resistant mice, implying changes in predicted functional capacity [38]. In contrast to a shift in gut microbiota composition during *P*. *yoelii* 17XNL infection, untargeted metabolomics showed modest alterations in metabolite profile of small intestine and ceca content and plasma during parasitemia except at peak parasitemia in both *P*. *yoelii* 17XNL hyperparasitemia-susceptible and -resistant mice [38].

### 4.2. Experimental cerebral malaria

Cerebral malaria is one of the most severe forms of malaria and a leading cause of malaria mortality in children (15% death rate) and adults (20% death rate) [57]. Nearly a quarter of cerebral malaria survivors suffer from life-long neurological sequelae and ongoing comorbidities [58]. Five studies have investigated the impact of experimental cerebral malaria (ECM) on gut microbiome composition in murine malaria [59–62] and one on upper gastrointestinal pathophysiology [63] with variable results. Taniguchi and colleagues (2015) and Shimada and colleagues (2019) reported intestinal pathology in C57BL/6 mice infected by *P*. *berghei* ANKA that develop ECM [62,63]. ECM caused weight loss, multiple red gastric patches, detachment of epithelia, gastric gas retention, enlargement of goblet cells, small intestine shortening, increased intestinal permeability, and caused dysbiosis [63]. Knowler and colleagues (2023) observed lengthening of small intestine in contrast to Shimada and colleagues (2019) [60,63]. *P*. *berghei* ANKA infection in C57BL/6 mice caused changes in gut bacteria composition with increased abundance of class *Clostridia;* family *Enterobacteriaceae*, *Verrucomicrobiaceae*; and genus *Akkermansia*, *Alistipes*, and *Alloprevotella* and decrease *Lactobacillaceae* family (Fig 2) [59,60,62]. Although *P*. *berghei* ANKA causes lethal ECM within 7 to 10 days, these *P*. *berghei* ANKA-induced changes in gut bacterial composition were long lasting if mice were treated with artemether [61].

### 4.3. Humans

There is limited information regarding the effect of malaria on gut microbiota composition in humans. Presently, 1 study has assessed this in the context of longitudinal analysis of stool bacteria compositions in Kenyan infants [64]. In this study, stool samples from infants (*n* = 10) from birth to 10 months were collected roughly 2 weeks before and 2 weeks after a febrile malarial episode and artemether-lumefantrine treatment. In contrast to model organisms, measurements of gut microbiota composition using multiple metrics of alpha and beta diversity did not show significant difference in structure and composition of gut microbiota [64]. That artemether-lumefantrine treatment had no effect on gut bacteria populations in the Kenyan infants, is consistent with a separate study in mice showing that artemether-lumefantrine and artesunate-amodiaquine treatment had no effect on gut bacteria communities [65]. There are important limitations to this study that must be considered including the small sample size of 10 infants, narrow age range of participants (<10 months old), lack of severe malaria, and lack of higher-resolution sample collection whereby gut bacteria could have changed and reverted to baseline. Therefore, additional longitudinal studies in humans that address these limitations and expand the geographical representation of participants are warranted to gain deeper insight. As other inflammatory and severe diseases have been shown to change gut bacteria compositions [66–68], it would not be surprising if severe malaria in children is indeed associated with changes in gut bacteria compositions.

### 4.4. Implications of malaria-induced gut microbiota changes

These observations raise an important question, what effect does *Plasmodium*-induced changes in gut microbiota composition have on current/subsequent malaria outcomes? The one study to assess this question performed FMTs from convalescent *P*. *yoelii* 17XNL (day 60 post infection) hyperparasitemia-resistant and -susceptible mice into germ-free mice. As noted above, *P*. *yoelii* 17XNL hyperparasitemia-resistant and -susceptible mice showed changes in gut bacteria communities following *P*. *yoelii* 17XNL infection, with decreasing differences in gut bacteria communities between these groups of mice at convalescence compared to differences observed pre-*P*. *yoelii* 17XNL infection [38]. Yet, the ex-germ-free mice colonized with gut microbiota from *P*. *yoelii* 17XNL hyperparasitemia-resistant and -susceptible mice were resistant and susceptible, respectively, to hyperparasitemia following *P*. *yoelii* 17XNL infection [38]. These results demonstrate that, at least within this model, *Plasmodium*-induced changes in gut bacteria composition do not change susceptibility to future *Plasmodium* infections. That *P*. *yoelii* 17XNL-induced changes in gut bacteria did not impact subsequent malaria outcomes is beneficial to human case-control studies, as it demonstrates the bacteria that are present prior to *Plasmodium* infection and cause susceptibility to severe malaria are not lost in the susceptible mice nor do these bacteria appear in the resistant mice. Therefore, even if *P*. *falciparum* causes changes in human gut bacteria populations, these data suggest that comparing children with asymptomatic *P*. *falciparum* infections compared to children with severe malaria has the potential to identify bacteria that contributed to the differential malaria outcomes.

## 5. Immunological mechanisms by which host microbiota can impact malaria outcomes

**Fig 3:**
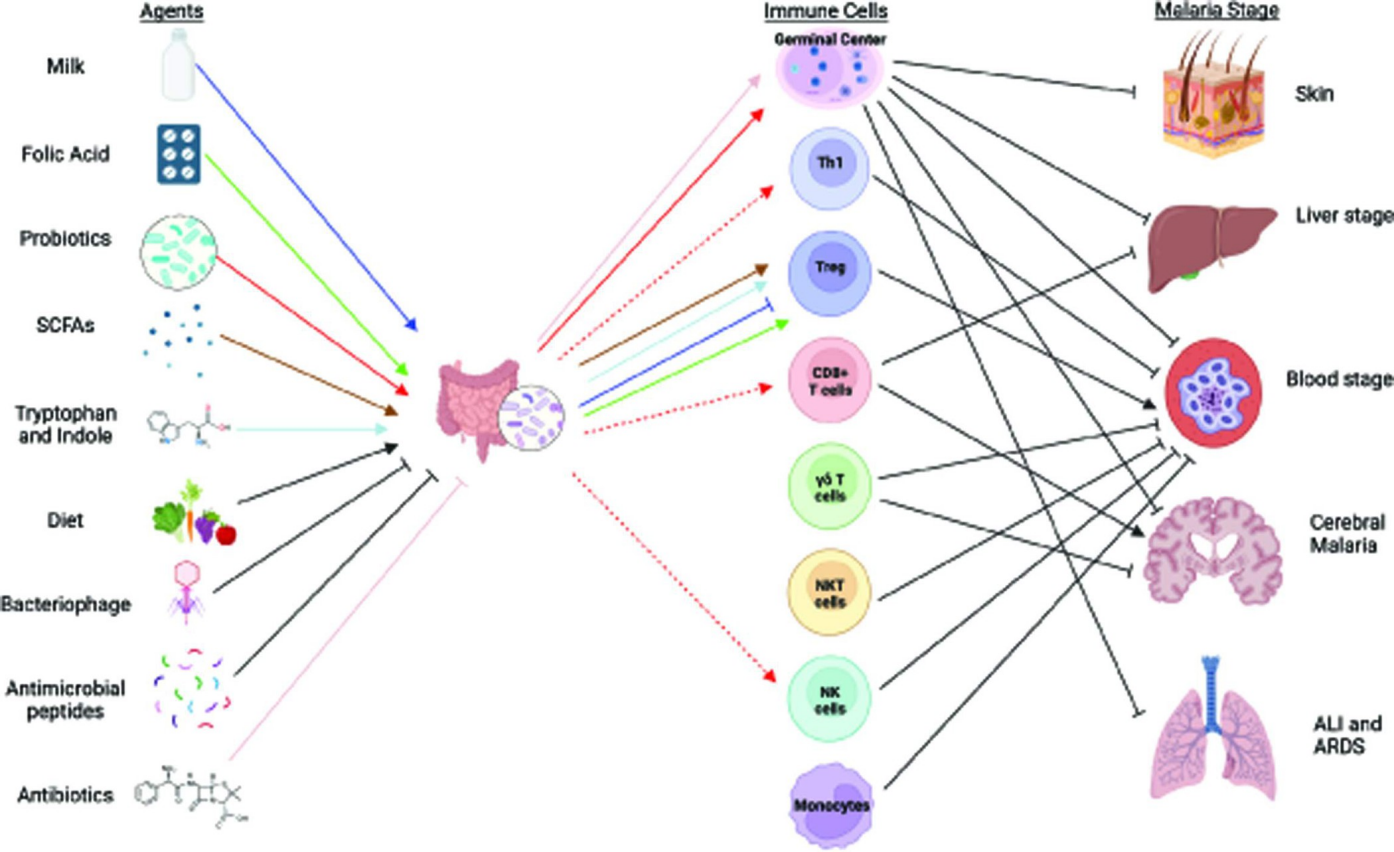
Gut microbiota intervention strategy and possible immunological mechanism of action against severity to malaria. Agents that can modulate gut microbiota composition or deplete gut bacteria that can influence gut or systemic immunity are shown. Dotted arrow indicates the potential interactions which need further validation. Color of arrows connect gut microbiota modifying agents and their impact on respective immune cells. Role of different immune populations to inhibit or exacerbate various stage and types of malaria are connected. Although, the exact mechanism on how gut microbiota impacts severe malaria is unknown, this figure provides a plausible connection between gut microbiota and malaria severity. Figure was created with BioRender.com.

Host immunity is essential to control *Plasmodium* infection [69], but immune responses are also involved in the pathology of malaria [70–73]. As such, it is crucial to understand the components of host immunity that both contribute to protection and pathogenesis in malaria and how these are regulated by gut microbiota. This knowledge has the potential to aid in the development of microbiome-based therapeutics to control *Plasmodium* infection and prevent life-threatening severe malaria. The contribution of innate and adaptive immunity during *Plasmodium* infection is reviewed elsewhere [74]. Contribution of specific gut microbiome in immunity and immune-mediated disorders and CD4+ T cell differentiation and function is published previously [9,75–77]. Here, we have reviewed components of the immune system that contribute towards control and pathogenesis of malaria and discuss specific gut microbiota that might affect these components of host immunity. Whether and how gut-derived immune cells impact the gut-distal immune response to malaria and other infections is yet to be fully understood (Fig 3).

### 5.1. GC B cells and TFh cells

GC reactions are required for production of high-affinity antibodies and long-term memory against malaria [69,78–80]. We have shown that gut microbiota compositions dynamically regulate the quality and quantity of GC reactions during *Plasmodium* infection in C57BL/6 mice infected with *P*. *yoelii* 17XNL [6,34]. Relatively higher abundance of *Clostridium papyrosolven*, *Alistipes putredinis*, *Alistipes timonensis*, and *Lactobacillus reuteri* at genus level and *Bacteroidiaceae* and *Lacnospiraceae* at family level were associated with susceptibility to hyperparasitemia while *Lactobacillus johnsonii*, among others, with resistance to hyperparasitemia correlating to GC responses in mice [6]. Possibly, gut microbiota associated with resistance to hyperparasitemia might be linked to higher levels of SCFAs, flagellin, peptidoglycans, lipopolysaccharides, cross reactive epitopes, but not PA in the gut, resulting in B cells in Peyer’s patches and the spleen producing better antibody responses [39,81,82].

### 5.2. T helper 1 (TH1) cells

The contribution CD4+ TH1 cells to protection against blood-stage malaria is complicated and nuanced [79]. Both IFNγ producing Th1 cells and IL-10 producing Th1 cells (Tr1) are required to control *Plasmodium* infection [83]. Tissue damage done by inflammatory cytokines like IFNγ is regulated by anti-inflammatory cytokine IL-10 [83,84]. Gut microbiota-derived SCFAs promote IL-10 production by Th1 cells to maintain intestinal homeostasis. Gut microbiota depleted with antibiotics have enhanced intestinal Th1 cell response [85], and C57BL/6 mice become resistant to severe hyperparasitemia to *P*. *yoelii* 17XNL infection when treated with oral antibiotics [6]. Additionally, probiotic *Lactobacillus* strains can result in macrophage-mediated induction of Th1 response [75,86].

### 5.3. Regulatory T cells

Foxp3+ Tregs have ubiquitous roles in anti-malarial immunity in both mice and humans, yet owing to the pleiotropic effect of these cells and the complexity of anti-malaria immunity, the full contribution of these cells to protection and pathology during malaria is not fully understood [69,87]. Lower Treg cell numbers are associated with lower parasite burden and better outcomes in humans during blood-stage malaria [72,88,89]. However, depletion of Treg cells in FoxP3–diphtheria toxin receptor (DTR) transgenic (DEREG) C57BL/6 mice did not decrease ECM severity suggesting a limited role in ECM [90]. Still, depletion of Tregs or blocking Cytotoxic T-lymphocyte-Associated protein (CTLA-4) expressed on Tregs during a narrow window-of-time just prior to peak parasitemia enhanced immune responses and accelerated parasite clearance. This enhanced protection was attributed to increased Tfh:B cell interactions in GC reactions generating a more robust antibody response during blood-stage *P*. *yoelii* 17XNL infection in mice [72]. Germ-free mice colonized with SFB have increased expression of RORγt^+^ Tregs [91], and SCFAs (e.g., butyrate and propionate) produced by commensal gut bacteria promote peripheral Treg generation [92]. Consistent with gut microbiota stimulating expansion of these cells, Tregs are significantly reduced in germ-free or antibiotic-treated mice [91]. Although the mechanism is unknown, mice treated with antibiotics have significantly decreased *P*. *yoelii* 17XNL burden compared to untreated mice [6].

### 5.4. CD8+ T cells

Cytotoxic CD8+ T cells can protect against liver-stage malaria while providing little help in controlling blood-stage malaria [69,93]. However, CD8+ T cells have been shown in rodent ECM to cause breakdown of the blood–brain barrier [94] and have been found in the brain microvasculature of humans [70,95,96]. Several studies have demonstrated the ability of gut microbiota to regulate CD8+ T cell responses. Mice that develop more colitis-associated tumors have increased numbers of CD8+IFNγ+ T cells in lamina propria with higher relative abundance of *Alistipes*, *Ruminococcus*, *Prevotellaceae*, and lower abundance of *Lachnospiraceae* in the gut compared to mice that develop low tumor burden [97]. In contrast, circulating numbers of gut-derived CD8+IFNγ+ T cells induced by a mixture of 11 gut bacterial species belonging to 8 genera (*Bacteroides*, *Parabacteroides*, *Alistipes*, *Paraprevotella*, *Eubacterim*, *Ruminococcaceae*, *Phascolarctobacterium*, and *Fusobacterium*) had enhanced anti-tumor immunity against subcutaneous engraftment of MC38 adenocarcinoma cell and anti-microbial immunity against oral *Listeria monocytogenes* challenge [98]. The ability of gut microbiota to modulate CD8+ T cell responses during malaria, in particular during cerebral malaria, is an area ripe for exploration.

### 5.5. γδ T cells

γδ T cells recognize *Plasmodium*-infected erythrocytes and destroy infected RBCs either through cytotoxic molecules or antibody dependent phagocytosis [99,100]. Additionally, liver-stage dependent, low blood-stage *Plasmodium* parasite mass activates γδ T cells to produce IL-17, which protects mice from lethal cerebral malaria by promoting erythropoiesis [101]. Administration of lactic acid producing *Lactobacillus plantarum* probiotic promotes hematopoiesis and erythropoiesis [102], whether lactic acid-producing bacteria confer protection against severe malaria remains unclear. Interestingly, C57BL/6 mice fed a high fat diet (HFD) are resistant to ECM [103]. Numerous factors may contribute to HFD protection to ECM, but a HFD can modulate gut microbiota, generally leading to a decrease in Bacteroidetes and increase in Firmicutes and Proteobacteria [104]. Mice fed HFD for 3 weeks have increased number of IL-17+ γδ T cells, IFNγ+ Th1 cells, and CD8+ T cells while decreased numbers of Tregs in the colon and small intestine [105]. However, increased abundance of Proteobacteria and lower abundance of *Clostridiaceae* and S24-7 is also associated with induction of CD4+ Tregs cells [106], highlighting the complex interaction between gut microbiota and immune system. Whether HFD protection from ECM is attributed to diet-dependent effects on gut microbiota and their modulation of host immune cells or alternative microbiome-independent effects is not known.

### 5.6. NKT cells

NKT cells have been shown to reduce blood-stage parasitemia due to enhanced secretion of IFNγ [107]. NKT cells are usually found in thymus, spleen, liver, and bone marrow [108]. Depleting gram-positive gut bacteria with vancomycin that are involved in the conversion of primary bile acids to secondary bile acids was able to induce hepatic NKT cell accumulation and decreased liver tumor growth [109]. Vancomycin-treated mice had negligible presence of *Bacteroidales* and significantly reduced *Clostridiales* [109]. Interestingly, monocolonization with *Clostridium scindens*, which can transform bile acid, was able to reduce hepatic NKT cells and recover *Bacteroidales* [109,110]. Yet, the impact of gut microbiota modulating NKT cells other than in the liver is unknown; therefore, the ability of gut microbiota to modulate NKT cells and impact blood-stage parasite burden is unknown. Presently, there are no reported effects of gut microbiota on *Plasmodium* liver-stage burden, but as NKT cells are believed to have a protective role in liver-stage *Plasmodium* infections [74,111], it raises the possibility gut microbiota may impact liver-stage burden via modulation of NKT cells.

### 5.7. Natural killer (NK) cells

NK cells have beneficial roles during *Plasmodium* infection [112]. NK cells produce inflammatory cytokines, kill infected RBCs, and participate in initiation and development of adaptive immune response during malaria infection [113–116]. Butyrate is one of the major SCFAs produced by gut microbiota that is reported to limit the effector function of human NK cells from blood in vitro by down-regulation of mTORC1 activity, c-Myc mRNA expression, and metabolism [117]. In contrast, dietary butyrate supplementation or treatment with *Clostridium butyricum* in mice treated with antibiotics early in life promoted the maturation and restored function of liver-resident NK cells [118]. *Faecalibacterium*, *Roseburia*, *Fusobacteria*, and *Eubacterium* are other bacteria that can produce butyrate [119]. Additionally, high salt diet can enhance NK cell functions in a gut microbiota dependent way by increasing the abundance of probiotic bacteria *Bifidobacterium* but increased gut permeability [120].

### 5.8. Monocytes

Monocytes have both protective and pathological role in malaria infection [121]. Enhanced p53 expression in monocytes are associated with attenuated *Plasmodium*-induced inflammation and protects from early fever during malaria infection in humans [30]. IFNγ leads to enhanced expression of p53 in monocytes to attenuate pro-inflammatory activation [30,122,123]. Gut microbial products have been associated with regulation and function of splenic monocytes [124]. Oral antibiotics treatment eliminated bacterial taxa from Bacteroides and Firmicutes along with other bacteria that reduced pattern recognition receptor ligands in the serum that led to immature phenotype of splenic Ly6C^high^ monocytes exhibiting decreased level of pro-inflammatory cytokines and increased phagocytic abilities [124].

## 6. Targeting gut microbiota as a strategy to decrease severe malaria

There are numerous approaches by which gut microbiota can be modulated. In this section, we have reviewed some of the past efforts and recent advances and strategies to manipulate gut microbiota. These approaches may serve as useful approaches to interrogate gut microbiota–host–parasite interactions, with some approaches serving as potential gut microbiota-based approaches to mitigate severe malaria. The latter will require extensive investigation and clinical trials with positive malaria outcome before any recommendations can be made (Fig 3).

### 6.1. High fat, calorie restricted, and low protein diets

Studies have shown that high fat, calorie restricted, and low protein diets are associated with favorable parasitemia and mortality outcomes in rodent malaria models [103,125,126]. However, the role of gut microbiota, mechanisms of action, and identification of targetable pathways are required to advance dietary gut microbiota-based therapeutics.

### 6.2. Milk

Rodents on a milk diet suppress *P*. *berghei* infection, while having no effect on *Nutallia rodhaini* (Babesia) or *Trypanosoma brucei* infection [127–129]. Similar protections were seen against 2 nonhuman primate (monkey) strains, *P*. *knowlesi* and *P*. *cynomolgi* [129]. Moreover, mice and monkeys fed a milk diet supplemented with p-aminobenzoate, a growth factor for many *Plasmodium* species, lost protection against malaria [129]. Milk consumption has been shown to decrease Bacteroidetes and *Prevotella* while increase Proteobacteria, *Bifidobacterium*, *Lactobacillus*, and *Roseburia* [130,131]. Connections between milk consumption and its effect on gut microbiota to modulate malaria severity warrants further investigation.

### 6.3. Folic acid (FA)

Controlled trials have found that FA supplementation compromised the efficacy of antimalaria drugs and should be avoided as supplement in children in malaria endemic regions [132,133]. FA induces Tregs [134] and production of folate is positively associated with higher relative abundance of *Bacteroides*, *Sutterella*, and *Parasutterella* [135] that are associated with high blood-stage parasite burden.

### 6.4. Probiotics

Probiotics are valued for the health benefits they confer upon the host, and these have been investigated in the context of *Plasmodium* infections. Heat killed *Lactobacillus sakei* HS-1 was able to minimize weight loss and mitigate intestinal pathology and limited small intestine shortening in C57BL/6 mice during *P*. *berghei* infection [136]. However, parasitemia load was not decreased in *Lactobacillus sakei*-treated mice [136]. Likewise, *L*. *casei* administration was shown to be protective against *P*. *berghei* as an adjunct therapy along with antimalarial treatment in BALB/C mice [137,138]. Additionally, C57BL/6 mice administered with *Bifidobacterium longum* alone had diminished *P*. *berghei* burden, colon inflammation, and significantly lower level of plasma TNFα and IFNγ compared to *B*. *longum* plus *L*. *casei* or *L*. *casei* alone [139]. Expression of intestinal CD103+ dendritic cells and intestinal Tregs were high in mice receiving *B*. *longum* [139]. CD103+ dendritic cells in nonlymphoid organs induces Tregs [140]. Dendritic cells have a critical role in initiating and regulating innate and adaptive immunity against malaria [141]. Finally, some probiotic bacteria strains express high levels of α-gal [142], which may facilitate protection against *Plasmodium* exoerythrocytic stages via induction of anti-α-gal antibodies, as discussed above.

One of the challenges with probiotics is poor engraftment. Understanding the dietary requirements of probiotic bacteria will be important as it can be targeted to improve engraftment. For example, some strains of *Bacteroides ovatus* can utilize the marine polysaccharide, porphyran, which is lacking in the vast majority of gut bacteria [143]. Transfer of the gene cluster for porphyran utilization from *B*. *ovatus* into another *Bacteroides* species lacking this cluster allowed fine tuning the engraftment of these bacteria into the competitive mouse gut microbiota niche when mice were provided porphyran in their diet. Similar approaches can be used to clone unique nutrient utilization gene clusters into probiotic strains with proven beneficial effect against malaria to overcome the probiotic wash-out effect.

### 6.5. Antimicrobial peptides

Non-immunogenic and non-toxic antimicrobial peptides from *Lactobacillus plantarum* strain LR/14 inhibited the growth of *P*. *falciparum* in vitro without any hemolysis [144]. *L*. *plantarum* can also inhibit many gram-positive and gram-negative bacteria [145], which can be exploited to manipulate gut microbiota composition.

### 6.6. Antibiotics

C57BL/6 mice treated with any of 4 antibiotics (ampicillin, gentamicin, metronidazole, and vancomycin) in drinking water prior to and during *P*. *yoelii* 17XNL infection significantly decrease the parasite burden. Of note, treatment of mice resistant to *P*. *yoelii* 17XNL hyperparasitemia showed no effect (i.e., the mice did not become susceptible). Moreover, treating with oral vancomycin 1 week prior to *P*. *yoelii* 17XNL infection, followed by cessation of treatment, significantly decreased *P*. *yoelii* 17XNL parasite burden. Intriguingly, preinfection vancomycin treatment afforded resistance to *P*. *yoeli*i 17XNL hyperparasitemia for at least 3 months post-cessation of vancomycin treatment. Although antibiotic use poses threat to emergence of antibiotic resistance, clinical trials performed with antibiotics (amoxicillin, cefdinir, ceftriaxone, metronidazole) were associated with positive outcome in malnourished children [146–148]. Thus, clinical trials to test antibiotics as an adjunct therapy to prevent and manage severe malaria may have merit.

### 6.7. Bacteriophages

Bacteriophages are viruses that can attack bacteria with specificity [149]. Bacteriophages, natural or engineered, can dynamically modulate gut microbiota and the metabolome [150]. Bacteriophages have been used to precisely modulate gut microbiota in diseases like IBD, colitis, type 2 diabetes, and colorectal cancer among others to improve prognosis with minimal damage to host gut microbiota [149,151,152]. Of course, this exciting technology comes with limitations and challenges [149]. For example, evolution of phage resistance, efficacy of phages against biofilms, and stability of phage preparations are a few important challenges [153]. Nevertheless, if specific gut bacteria are involved in susceptibility to severe malaria, then targeting these bacteria via bacteriophages might result in promising outcomes.

### 6.8. Short chain fatty acids (SCFAs)

Previously, we have shown that levels of SCFAs like propionic acid, butyric acid, and valeric acid were significantly different among mice susceptible and resistant to *P*. *yoelii* 17XNL hyperparasitemia [39]. Culminating evidence suggests important roles of SCFAs in the development and regulation of the immune system and gut barrier integrity that impact disease severity and enhanced health [154,155]. Consequently, SCFAs may impact malaria severity; however, the contribution of any specific SCFAs in malaria severity is yet to be studied.

### 6.9. Dietary tryptophan and indoles

Gut microbiota conversion of dietary tryptophan to indoles has important roles in enhancing gut barrier integrity and binds to AhR receptor on immune cells to induce anti-inflammatory and antimicrobial properties [156,157]. The indole moiety is one of the most promising chemotypes for the development of antiparasitic drugs [158]. Indole-3-acetic acid activated AhR pathway promotes anti-inflammatory cytokine IL-10 and up-regulated Foxp3 and increased Treg cells in a proteoglycan (PG)-induced ankylosis mouse model [159,160]. Therefore, the role of gut-derived indoles and the ability of indoles to induce Tregs in malaria will be an interesting avenue of research [161,162].

## 7. Conclusion

Within the last 2 decades, our understanding of gut microbiota function has increased exponentially due to advances in high-throughput omics technologies like nucleic acid sequencing, metabolomics, metatranscriptomics, proteomics, interbacterial and intrabacterial interaction within the host, interdisciplinary studies, and the rise of artificial intelligence and machine learning in biological sciences. With this new knowledge, the scientific community is positioned to identify specific gut microbiota and microbiota-derived products and their interactions with the host immune system in modulating the severity of malaria. The capacity to precisely identify these biomarkers will increase with continued development and innovation in the microbiome field. With these advancements, gut microbiome-based therapies may one day be used to mitigate the severity of malaria associated with *Plasmodium* infection.
